# The Morphological Identity of Insect Dendrites

**DOI:** 10.1371/journal.pcbi.1000251

**Published:** 2008-12-26

**Authors:** Hermann Cuntz, Friedrich Forstner, Juergen Haag, Alexander Borst

**Affiliations:** 1 Wolfson Institute for Biomedical Research and Department of Physiology, University College London, London, United Kingdom; 2 Department of Systems and Computational Neurobiology, Max-Planck Institute for Neurobiology, Martinsried, Germany; University College London, United Kingdom

## Abstract

Dendrite morphology, a neuron's anatomical fingerprint, is a neuroscientist's asset in unveiling organizational principles in the brain. However, the genetic program encoding the morphological identity of a single dendrite remains a mystery. In order to obtain a formal understanding of dendritic branching, we studied distributions of morphological parameters in a group of four individually identifiable neurons of the fly visual system. We found that parameters relating to the branching topology were similar throughout all cells. Only parameters relating to the area covered by the dendrite were cell type specific. With these areas, artificial dendrites were grown based on optimization principles minimizing the amount of wiring and maximizing synaptic democracy. Although the same branching rule was used for all cells, this yielded dendritic structures virtually indistinguishable from their real counterparts. From these principles we derived a fully-automated model-based neuron reconstruction procedure validating the artificial branching rule. In conclusion, we suggest that the genetic program implementing neuronal branching could be constant in all cells whereas the one responsible for the dendrite spanning field should be cell specific.

## Author Summary

Neural computation has been shown to be heavily dependent not only on the connectivity of single neurons but also on their specific dendritic shape—often used as a key feature for their classification. Still, very little is known about the constraints determining a neuron's morphological identity. In particular, one would like to understand what cells with the same or similar function share anatomically, what renders them different from others, and whether one can formalize this difference objectively. A large number of approaches have been proposed, trying to put dendritic morphology in a parametric frame. A central problem lies in the wide variety and variability of dendritic branching and function even within one narrow cell class. We addressed this problem by investigating functionally and anatomically highly conserved neurons in the fly brain, where each neuron can easily be individually identified in different animals. Our analysis shows that the pattern of dendritic branching is not unique in any particular cell, only the features of the area that the dendrites cover allow a clear classification. This leads to the conclusion that all fly dendrites share the same growth program but a neuron's dendritic field shape, its “anatomical receptive field”, is key to its specific identity.

## Introduction

Dendrite morphology is the most prominent feature of nerve cells, typically used by neuroanatomists to discriminate and classify them [1]. These tree-like ramifications represent the input region of the neurons and fulfil the role of a complex computational unit [2]–[5]. Typically, dendritic arborizations are analyzed in a descriptive way, e.g. by enumerating local and global branching parameters [6]–[8]. Very little is known about the general rule leading to their distinct appearance partly due to the wide variety among different neurons. In insects, same neurons across individuals are rather invariant in their anatomy and constant in their function. Lobula plate tangential cells (LPTCs) of the fly visual system [9] are uniquely identifiable and are therefore ideal subjects for investigating the basic rule constraining dendrite formation. They integrate local motion information over an array of retinotopically arranged columnar elements [10]. Accordingly, their planar dendritic trees cover the area corresponding to their distinct primary receptive fields. In this paper we isolate potential fundamental principles which may lead to the morphological identity of individual LPTCs.

## Results

**Figure 1:**
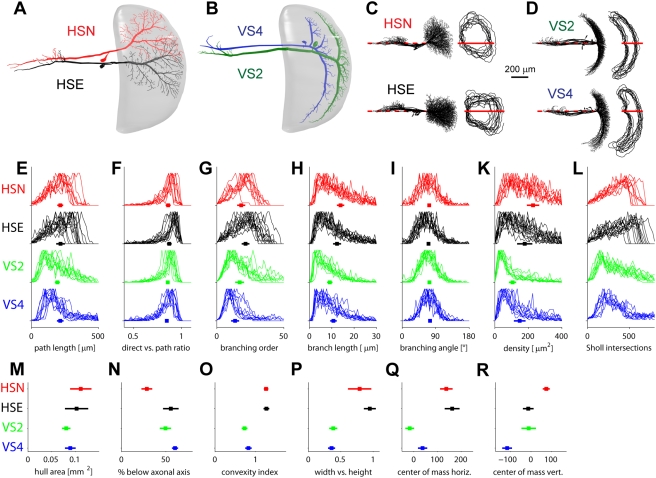
Dendrite morphological statistics. (A,B), Sketches showing HSE and HSN (A) and VS2 and VS4 (B) in the context of the lobula plate (gray). (C,D), Superimposed full anatomies of all individual cells sorted according to their respective cell type. Cells were aligned along their axonal axis (red lines). To the right, the corresponding dendrite spanning fields are outlined. (E–K) Statistics specifically related to dendrite branching. Statistics are represented as superimposed distribution histograms, filled squares show mean values and error bars correspond to standard deviation between individual dendrites: (E) path length to root values for all topological points; (F) ratios between direct and path distances from each topological point to the dendrite root; (G) topological point branching order values, a measure for the topological distance from the dendrite root; (H) length values of branch pieces between topological points; (I) branching angle values at all branching points between the two direct daughter branches within the plane in which they lay; (K) surface area values assigned to each topological point after Voronoi segmentation indicating topological point density and distribution homogeneity. (L) Sholl intersection plots: number of intersections of each tree with circles with increasing diameter. (M–R) Statistics describing the dendrite spanning field: (M) total surface value of spanning field; (N) percentage of the spanning field below the axonal axis; (O) convexity index of the spanning field; (P) ratio of width against height of the spanning field; (Q and R) horizontal and vertical coordinates of centre of mass of the dendrite spanning field.

We studied inter-individual constancy and variability in four members of the LPTC group: the equatorial and the northern cell of the horizontal system (HSE and HSN, Figure 1A), and two members of the vertical system (VS2 and VS4, Figure 1B), each of them represented by at least ten individuals from different flies. Two-photon image stacks were acquired from cells filled with fluorescent dye in the living blowfly, *Calliphora vicina*. Subsequently, the anatomy of each neuron was manually traced and described by a set of connected cylinders (see detailed explanation on the reconstruction procedures in the Methods section). The idea was, in concordance with previous publications [6]–[8], to use statistical distributions over morphological parameters thereby isolating key features of dendritic branching. Next to classical branching parameters on the “topological points” (branching and termination points in the tree) such as branching order and path lengths to the root, we parameterized the area covered by the dendritic tree, the so-called “dendrite spanning field” [11]. We defined spanning field by drawing a contour around the dendrite at a distance of 25 µm after orienting the reconstructed neuron along its axonal axis (Figure 1C and 1D).

**Figure 2:**
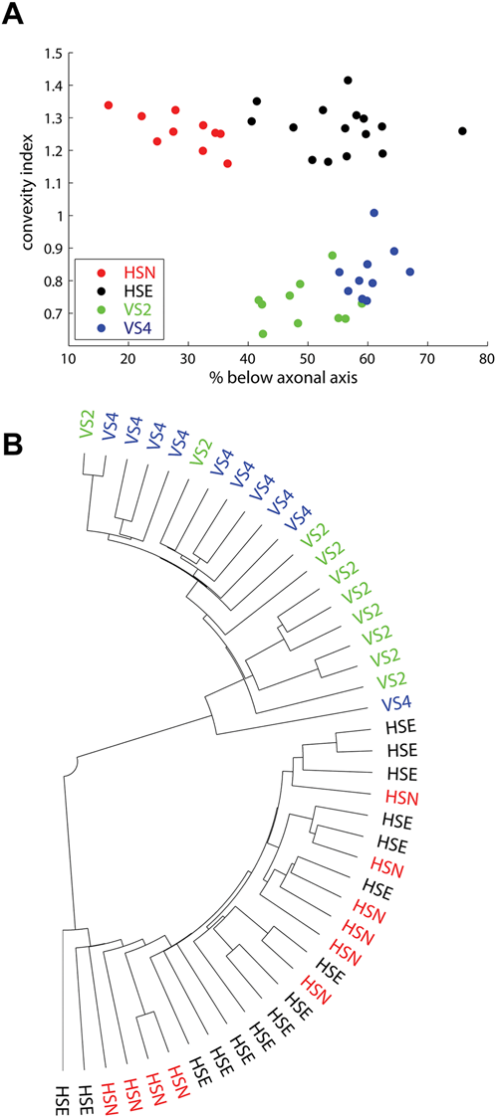
Cluster analysis. (A) Dendrite spanning fields are readily separable into the individual cell types at the example here of two parameters only: the convexity and the relative location to the axonal axis (B) Cluster analysis using three parameters of a generalized extreme value distribution fits for branching properties from Figure 1E, 1G, and 1K.

Regarding branching-specific statistics (Figure 1E–K), qualitative distinction was possible only by detailed examination of distributions of topological point density, path length to the root and branch order. Ratios between direct and path distances of the root (Figure 1F) followed a narrow distribution close to 1 in all cases for all topological points. Path length histograms (Figure 1E) therefore corresponded to the Sholl intersection diagram (Figure 1L), a measure typically used to describe branching topology. On the other hand, parameters relating to the spanning field plainly reflected cell type specific differences: All four cells could be readily discriminated by eye by their relative position and the shape of their dendrite spanning fields (Figure 1C and 1D, parameters see Figure 1M–R). Those differences were in conformity with the respective primary receptive field locations in the retinotopic arrangement. HS and VS spanning fields were easily distinguished by either their convexity index (Figure 1O) or the ratio of width against height (Figure 1P). Finer differentiation of HSE against HSN and VS2 against VS4 was provided directly by their relative location to the axonal axis (Figure 1N), and accordingly by their centre of mass (Figure 1Q and 1R). We investigated the descriptive power of spanning field parameters versus branching parameters in a quantitative way (Figure 2). Spanning field related parameters readily grouped individual cells into their respective cell types as shown simply by plotting convexity index values against the contextual relative location off the axonal axis (Figure 2A). On the other hand, even a highly-dimensional clustering analysis on the basis of parameterized shape fits of the distributions in Figure 1E–K (see Figure S2) or subsets of these did not allow the separation of the real cells into their respective groups. Best clustering was obtained using path length, density and branching order distributions which separated HS from VS cells but not the members of the two families (Figure 2B). In accordance to these findings we postulated that if the spanning area best determines neuronal appearance, the particularities in branching parameter distributions might be merely a consequence of the neuronal target zone.

**Figure 3:**
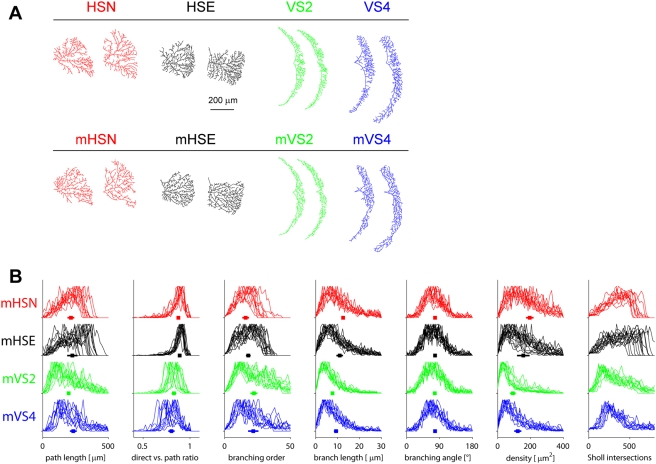
Artificial dendrites grown in real dendrite spanning fields. (A) Artificial dendrites: two examples of each cell type. Upper row: real dendrites. Lower row, marked by preceding “m”: artificial dendrites corresponding to each of the spanning field. (B) Artificial dendrite parameter distributions as in Figure 1E–K showing the similarity to their real counterparts.

In order to identify the critical impact of spanning field shape on branching parameters, artificial dendrites were constructed covering the same region. Inside the contours of the original cells, random points were distributed following their respective density map. An iterative greedy algorithm was launched starting at the coordinates of the real dendrite root. At each step, a connection was added from the existing tree to one of the unconnected random points according to a cost function which kept house of both total amount of wiring and total path length from the root to each point [12]. The number of random points was set to match the resulting number of topological points with the original dendrites. Improved appearance and overall path distance to the root was achieved by a subsequent smoothing step along primary branches (see Methods section). This resulted in artificial dendrites confined to the same area as the corresponding *in-vivo* dendrite reconstructions which were virtually indistinguishable from their real counterpart (Figure 3A; see Figure S3 for a full overview and Video S1 depicting the constructing process). Interestingly, artificial dendrites also yielded quantitatively similar parameter distributions in all cases (Figure 3B, compare with Figure 1E–L). The exact same branching rule can therefore account for all individual morphologies after constraining the spanning field shape alone.

**Figure 4:**
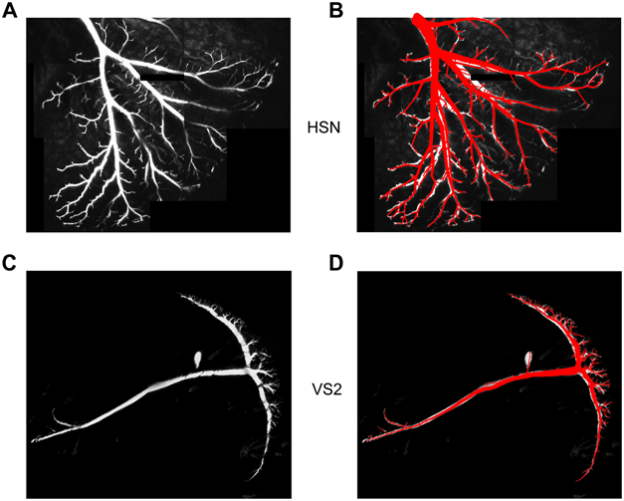
Model-based reconstruction of neuronal branching from 3D two-photon image stacks. Depicted at the example of an HSE dendrite (A,B) and of a VS2 cell (C,D). Left, maximum intensity projections of the image stacks containing fluorescent cells. Right, overlaid reconstructed branching in red.

Consequently, one could consider that original raw fluorescent images containing a labelled neuron would correspond to a distribution of interconnected points within a spanning field. Then, if our assumptions about the branching rule are correct, one should be able to apply it to obtain the branching model directly from the image material. We therefore applied the same greedy algorithm describing our branching rule for artificial dendrites on structural points extracted from the raw data via image skeletonization. The results of such an attempt are shown at the examples of an HSE dendrite (Figure 4A and 4B) and a full VS2 cell (Figure 4C and 4D). Faithful cylinder models of almost all branches could be retrieved in a fully automatic way from the image material after simply assigning manually a starting location at the dendrite root (see detailed description of the procedure in the Methods section).

## Discussion

In summary, we claim that all cells analysed here follow the same branching rule, and that their morphological identifier is the shape of their dendrite spanning fields. This claim is supported by the presented branching statistics, the previously proposed branching rule [12] and its reapplication in a heuristic reconstruction algorithm. Early approaches to describing and reconstructing dendrite branching in general had failed to take into account a major functional constraint governing dendrites: their need to reach specific input locations. More recent attempts to constructing dendrite morphology in relation to their function and the location of their inputs had led to dendrite structures of low complexity and accuracy in spite of high computational costs [13],[14]. However, circuitry and connectivity as well as simple wire packing issues are known to be determinants of dendrite morphology [15],[16]. In addition, the specific organization and architecture of many parts of the brain helps to reduce wiring costs for the circuitry [17],[18]. It is therefore not surprising that such constraints can be used to describe dendrite branching in LPTCs and other cells. Other planar space-filling cells, the cerebellar Purkinje cells, certainly follow a similar rule [19]. However, the suggested approach is not restricted to planar dendrites and future analysis will cover all different neuron arborizations to clarify the ubiquity of the suggested branching rule. At the example of LPTCs, the usefulness of the approach presented here can be put forward: LPTC electrophysiology was studied in great depth e.g. [20] and precise models, so-called compartmental models, including the detailed anatomical structure were designed and are continuously being improved [21]–[24]. Understanding LPTC branching, these constraints can be directly put in relation with the optic flow processing occurring within their circuitry [20],[23]. Assuming generality of principles, even the function of cells, which have not yet been reconstructed, can be inferred based on the contours of their dendrites alone. Moreover, the fly is the model animal in which the molecular components that determine neural growth are currently being unveiled, mainly through genetic tools [25],[26]. Our framework therefore allows a quantitative study of the impact of gene modifications far beyond basic statistics. In particular, molecular principles guiding neuronal self-avoidance during development [27] and others can now be put in relation with the branching constraints presented here. Eventually, studying molecular factors shaping dendritic spanning fields separately from a specific branching rule within should elucidate a fundamental organizational element in the brain, i.e. the neuron's branching structure.

## Methods

### Reconstructions

Female blowflies (*C. vicina*) were dissected as described in [28]. In each subject either one or two different HS or VS cells were filled with a fluorescent dye (Alexa 488). Flies were viewed under a custom built two-photon microscope [29], orienting the planar cells as orthogonal as possible in respect to the laser beam to minimize the amount of images in the Z-direction. In order to capture the entire expansion of the cells, 6 to 15 adjacent stacks (210 µm×210 µm area in XY x ∼30 in 2 µm Z-steps) were taken from different XYZ positions with an overlap of about 10 percent (Figure S1A). The image stacks were then transferred to Matlab (Mathworks, Natick, MA) and all further analysis was performed there in custom written software. Manual fine tuning of the original coordinates from the individual stacks was usually necessary to obtain a precise alignment in three dimensions. Maps of maximum intensity and corresponding depth were computed along the Z-axis. This reduction from 3D-data to two 2D images was sensible as there were no or very few 3D crossings of branches and all cells were planar. Based on these images cylinder models of the branching structure were obtained in a semi-automated way: interactive software allowed switched viewing of either Z-projection or an individual slice of an image stack (Figure S1B). The widths of 2D rectangles connecting the end points were fitted by gauss functions to suggest a diameter for the cylinders (Figure S1D). Z-values were attributed to each cylinder directly from the depth-map according to their 2D location. Quick tracing results (30 min) were achievable working with maximum Z-projections alone, although slight movements of the living fly compromised the accuracy of the projection image (Figure S1C). In order to achieve a higher accuracy, some manual corrections based on individual slices were necessary in all reconstruction steps. Taking advantage of the planar cell morphology allowed quicker reconstructions compared to other approaches [30]: detailed cell models with about 700 to 1600 compartments were obtained typically within around 2 hours. Jumps in the Z-axis were smoothed by use of linear interpolation. Internally and externally, the models were stored in the SWC format [31]. The reconstructions can be downloaded at: (http://www.neuro.mpg.de/english/rd/scn/research/ModelFly_Project/downloads/)

### Dendrite Statistics

For simplification, the resulting generic directed graphs were transformed into strict binary trees by substituting multifurcations with several bifurcations after minimally shifting the branches on their parent cylinder. Region indices [32] (soma (1), axon (2) or dendrite (3)) were manually attributed written to the SWC file. The somata in all cells consisted of a clearly separated bag-like structure that branched from the axon or dendrite. The last branch point (very short branches were ignored) before the soma was chosen to be the end of the dendrite and the beginning of the axon. The dendrite root was set to the primary branching point. Axonal parameters showed no trend to classify the cells (data not shown). There was no obvious correlation between axonal and dendrite length measures. Hence, size normalization of the cells was discarded. Dendrite flattening was performed as a morphometric transform [33] (Figure S1E). A distance isoline to any point on the dendrite was drawn at a 25 µm threshold to determine the dendrite spanning fields (Figure S1F). This corresponds to performing a morphological dilation on the same points with a 25 µm radius disc. For most statistics, only the branching and termination points ( = topological points) were selected as the carrier points for the topological complexity. A Voronoi segmentation was performed on these points in order to express space-filling density distribution (Figure S1G, used in Figures 1K and 3B). The density value therefore describes the area in vicinity of a specific branching or termination point. All LPTC reconstructions were rotated in order for both the dendrite root and the furthest axon terminal tip to lie on the horizontal line building the axonal axis. In order to obtain a measure for the convexity of dendrites, the convex hull was drawn around all dendrite nodes. The surface ratio between the dendrite spanning field (see above) and this convex hull was chosen as a characteristic spanning field parameter, the convexity index (Figure 1O). Centre of mass was calculated by taking the mean horizontal and vertical values of the line surrounding the dendrite spanning field (Figure 1Q and 1R).

### Cluster Analysis

Clustering (Figure 2) was done on the three parameters which enabled a by eye discrimination of VS and HS cells in Figure 1: the branching order, the path length and the density. Their histograms were collapsed to three values (mean, standard deviation and shape parameter) by fitting them to a generalized extreme value distribution (Figure S2). After normalizing to weigh parameters equally, Euclidean distances between the different dendrites in the resulting 9 dimensional parameter space were clustered hierarchically using the single linkage algorithm and displayed as dendrograms. As an alternative, the principal components of the matrix containing the normalized scalar parameters for each tree were obtained and the trees observed in the corresponding reduced dimensionality plot: no better grouping was possible with this method (data not shown).

### Artificial Dendrites

Boundary-corrected density maps of dendrite topological points were derived from real cell dendrites (Figure S1H–L). Random points were distributed according to the obtained density maps (Figure S1M). An extended greedy minimum spanning tree algorithm [12] was applied on these points starting at the root point of the original dendrite (Figure S1N). The number of random points was increased until the resulting number of topological points in the artificial dendrites matched the original dendrites. XY-coordinates of points on longer branches were smoothed by Spline interpolation to result in realistic dendrites (Figure S1O). Similar conclusions would arise if artificial dendrites were constructed on random points distributed entirely homogeneously (data not shown).

### Automatic Reconstruction

3D image stacks from one HSE dendrite and a full VS2 cell were submitted to 2D anisotropic filtering, morphological closure and subsequent brightness level thresholding. After 3D skeletonization and sparsening the carrier points, the remaining points were submitted to the same greedy algorithm (started at a user defined dendrite root location) as used for obtaining artificial dendrites Quadratic diameter decay was mapped on the resulting trees [12] (see Figure S1P).

## Supporting Information

Figure S1 Sketches describing the manual cell reconstruction process and the subsequent handling of dendrite morphology. (A) Assembled maximum Z-Projection of an HSN with ten overlapping image stacks. (B) Example of a reconstructed sub-tree of an HSN cell superimposed on a single slice from one image stack. (C) Compromising effects of the maximum Z-Projection (right) compared to the original slice (left, arrows show loss of branches). (D) Examples of automatic diameter approximations. Normalized positions 0.25, 0.5 and 0.75 on the midline and 40 half pixels in orthogonal direction were used to construct a sampling grid that covers a branch's thickness (first panel). The average over the resulting sampling matrix was convolved with the first derivative of a Gaussian distribution (little box) to emphasize brightness changes. The diameter was obtained by the distance from the centre of the maximum plateau in the mean signal to the null in the derivative of the convolved signal. (E) Planar dendrites were mapped entirely to two dimensional space (black original, red flattened dendrite). (F) The dendrite spanning fields were determined by drawing a region at 25 μm away from any point on the dendrite. (G) Topological point density distribution was obtained by Voronoi segmentation (green borders) with a dendrite spanning field boundary. Shaded gray scale indicates surface area of Voronoi pieces. Overlaid dendrite in red. (H–P) Steps in the creation of artificial dendrites: (H) dendrite topological points were morphologically closed (dilation followed by erosion) with a 25 μm radius disc and the resulting binary image smoothened with a Gaussian filter of 25 μm variance; (I) This was then cut out by the boundaries of the closed image, representing for each location in the dendrite spanning field the error made when smoothly averaging the density; (K) density estimation of topological points by Gaussian filtering with a 25 μm variance. (L) the density map in (K) was normalized by the estimation error obtained in (I); (M) random points (green) were distributed according to the corrected density distribution with sharp boundaries; (N) preliminary artificial dendrite following the iterative greedy algorithm presented previously [12] on green points in (M); (O) Artificial dendrite after smoothing along heavier branches; (P) quadratic diameter decay was mapped on the resulting dendritic structure according to an optimization of synaptic democracy [12]. The resulting artificial dendrite shows similarity with the original tree in (F). In (H–L) the flattened original dendrite from (F) was overlaid on top of the respective gray-scale maps.
(4.55 MB TIF)

Figure S2 Supplementary information on cluster analysis. (A) Generalized extreme value fits for the distributions shown in Figure 1E–K. This approach allowed obtaining three parameters for each distribution. These were used for the cluster analysis in Figure 2B and here. (B) Full cluster analysis for all models presented in the article. The method applied corresponds to Figure 2B of the article. Dendrites of individual cells were tagged by index numbers. Additionally, artificial dendrites from Figure 3 marked with a preceding “m” and lighter colours were included. Artificial dendrites mingled with their corresponding real counterparts indicating that they were similar to real cells in respect to their branching rule. Automatically reconstructed dendrites from Figure 4 were included marked by a preceding “--------r”. (C) Spanning field parameters as in Figure 2A but including the automatically reconstructed VS2 and HSN cells marked by a star. Line connects the automatically reconstructed dendrites with the corresponding manual reconstructions.
(1.20 MB TIF)

Figure S3 Overview of all 45 reconstructed LPTC dendrites and their artificial correlates. (A) Real manual dendrite reconstructions. (B) All constructed artificial LPTC dendrites. The model dendrites were grown in the spanning fields displayed in (A) in the same order. Diameter tapering was mapped here onto the branching structures for visual aesthetic purposes [12]. The artificial dendrites are hard to distinguish from their biological counterparts. (C, D) Overview of all dendrograms: comparison between reconstructed and artificial dendrites same colours as used in the main article. Dendrograms were sorted to put heavier trees (with larger sub-trees) on the left side. Although, taken one by one, dendrograms of artificial dendrites would not perfectly reproduce the corresponding partner (since they originated from random distributions of points), a trend of similarity is evident. This particularly illustrates how the differences in branching between HS and VS cells relates to the spanning fields of their dendrites since all artificial dendrites originate from the same branching rule. This is strongly in favour of a common branching rule for all cells. And this common rule is most likely very similar to the one applied to obtain the artificial dendrites.
(2.02 MB TIF)

Video S1 Demonstration of the artificial growth process. Dark red axonal arborizations are randomly distributed and correspond to target points. Iteratively, unconnected points are added to the tree (green). At each time step, for visual purposes, diameter tapering was mapped onto the tree as in Figure S1P and the existing tree was smoothed. These two steps were really performed after the entire growth in the artificial dendrites used in this paper. The artificial dendrite shown here is based on the HSN cell of Figure S1.
(2.77 MB AVI)
