# Supplementary figures and images for: The Morphological Identity of Insect Dendrites
Source: PLoS Comput Biol. 2008 Dec 26;4(12):e1000251. doi: 10.1371/journal.pcbi.1000251 (PMC2588660; doi:10.1371/journal.pcbi.1000251)

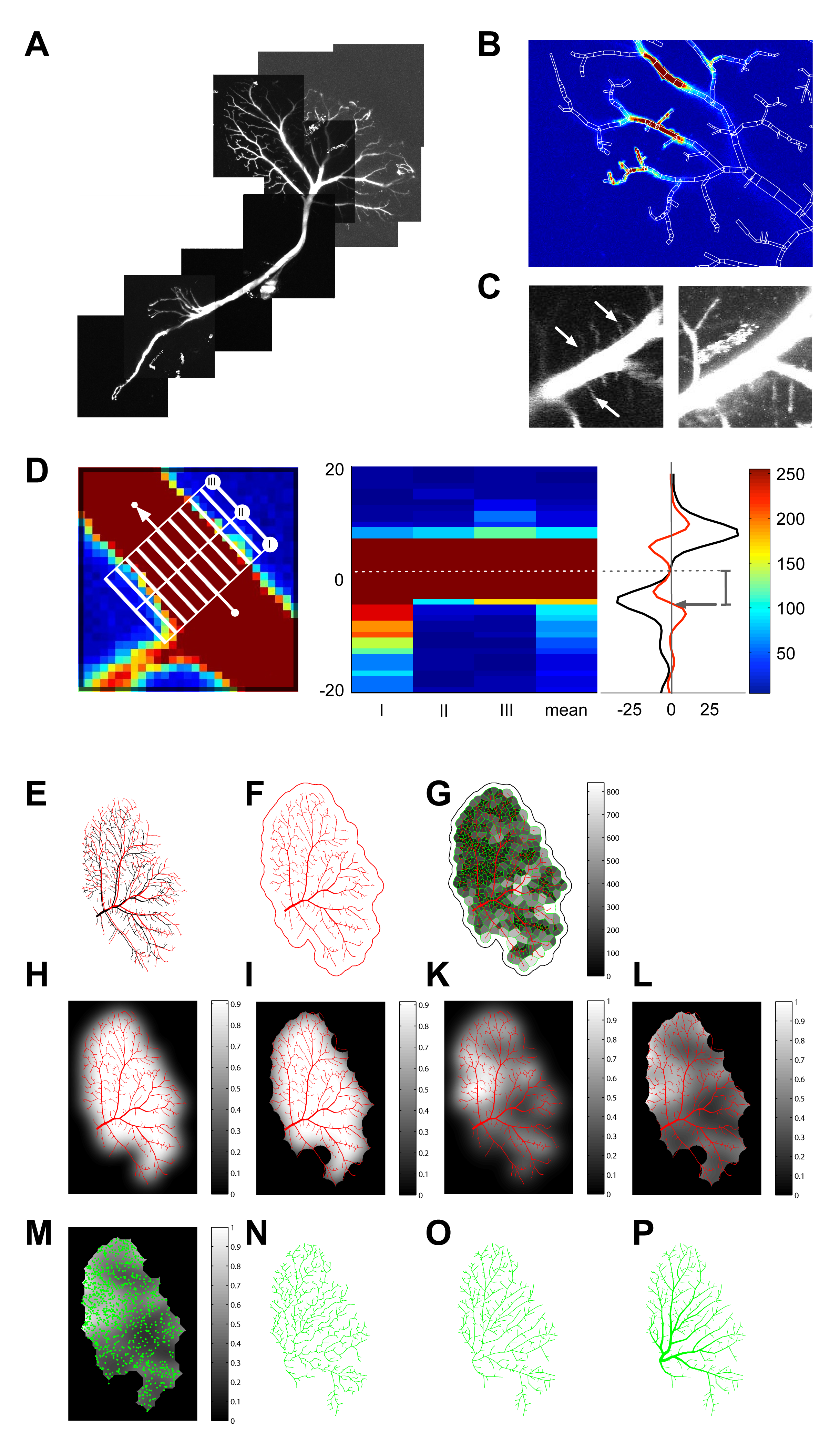

Supplement: Figure S1 — Sketches describing the manual cell reconstruction process and the subsequent handling of dendrite morphology. (A) Assembled maximum Z-Projection of an HSN with ten overlapping image stacks. (B) Example of a reconstructed sub-tree of an HSN cell superimposed on a single slice from one image stack. (C) Compromising effects of the maximum Z-Projection (right) compared to the original slice (left, arrows show loss of branches). (D) Examples of automatic diameter approximations. Normalized positions 0.25, 0.5 and 0.75 on the midline and 40 half pixels in orthogonal direction were used to construct a sampling grid that covers a branch's thickness (first panel). The average over the resulting sampling matrix was convolved with the first derivative of a Gaussian distribution (little box) to emphasize brightness changes. The diameter was obtained by the distance from the centre of the maximum plateau in the mean signal to the null in the derivative of the convolved signal. (E) Planar dendrites were mapped entirely to two dimensional space (black original, red flattened dendrite). (F) The dendrite spanning fields were determined by drawing a region at 25 μm away from any point on the dendrite. (G) Topological point density distribution was obtained by Voronoi segmentation (green borders) with a dendrite spanning field boundary. Shaded gray scale indicates surface area of Voronoi pieces. Overlaid dendrite in red. (H–P) Steps in the creation of artificial dendrites: (H) dendrite topological points were morphologically closed (dilation followed by erosion) with a 25 μm radius disc and the resulting binary image smoothened with a Gaussian filter of 25 μm variance; (I) This was then cut out by the boundaries of the closed image, representing for each location in the dendrite spanning field the error made when smoothly averaging the density; (K) density estimation of topological points by Gaussian filtering with a 25 μm variance. (L) the density map in (K) was normalized by the es [file pcbi.1000251.s001.tif]

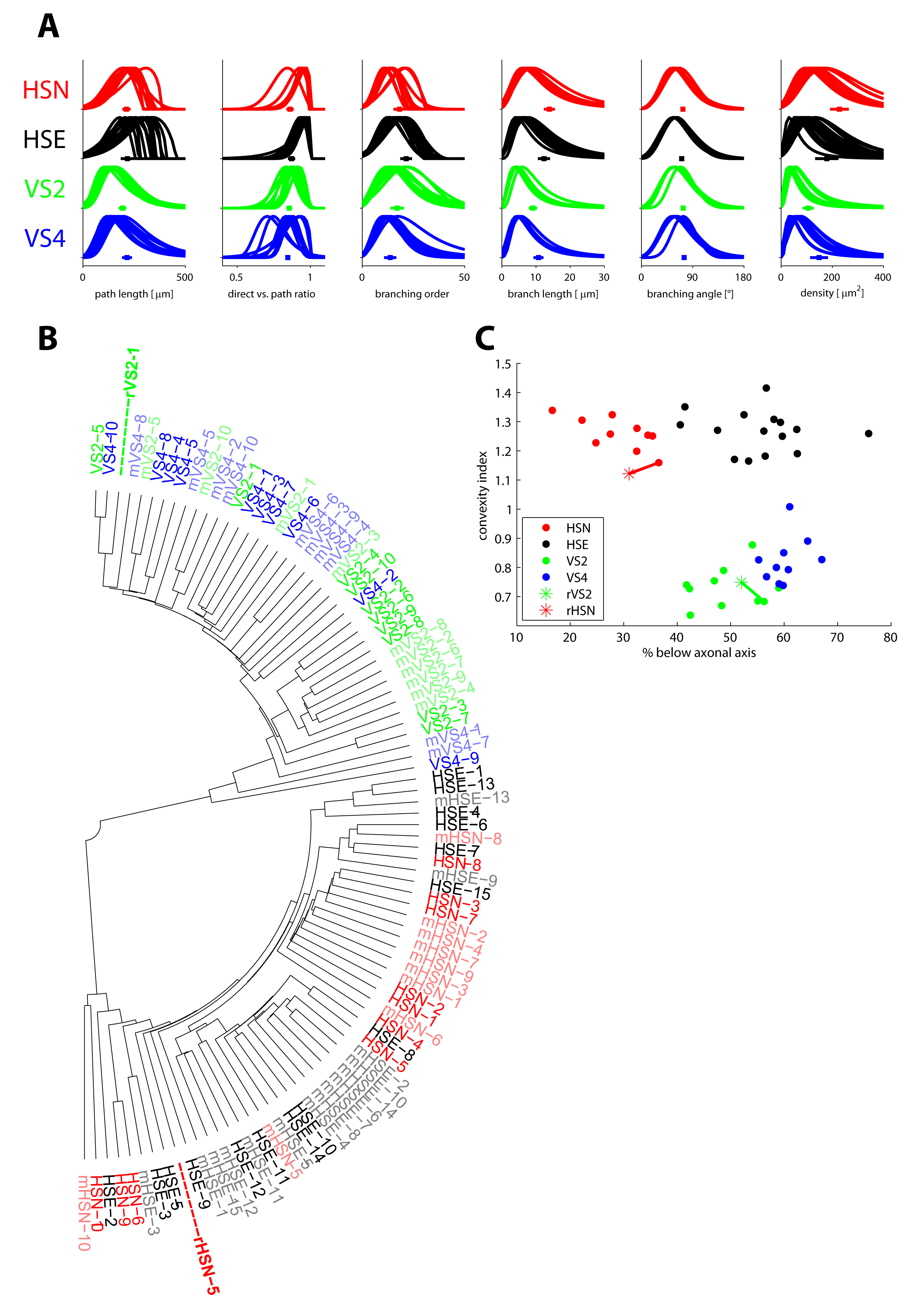

Supplement: Figure S2 — Supplementary information on cluster analysis. (A) Generalized extreme value fits for the distributions shown in Figure 1E–K. This approach allowed obtaining three parameters for each distribution. These were used for the cluster analysis in Figure 2B and here. (B) Full cluster analysis for all models presented in the article. The method applied corresponds to Figure 2B of the article. Dendrites of individual cells were tagged by index numbers. Additionally, artificial dendrites from Figure 3 marked with a preceding “m” and lighter colours were included. Artificial dendrites mingled with their corresponding real counterparts indicating that they were similar to real cells in respect to their branching rule. Automatically reconstructed dendrites from Figure 4 were included marked by a preceding “--------r”. (C) Spanning field parameters as in Figure 2A but including the automatically reconstructed VS2 and HSN cells marked by a star. Line connects the automatically reconstructed dendrites with the corresponding manual reconstructions. (1.20 MB TIF) [file pcbi.1000251.s002.tif]

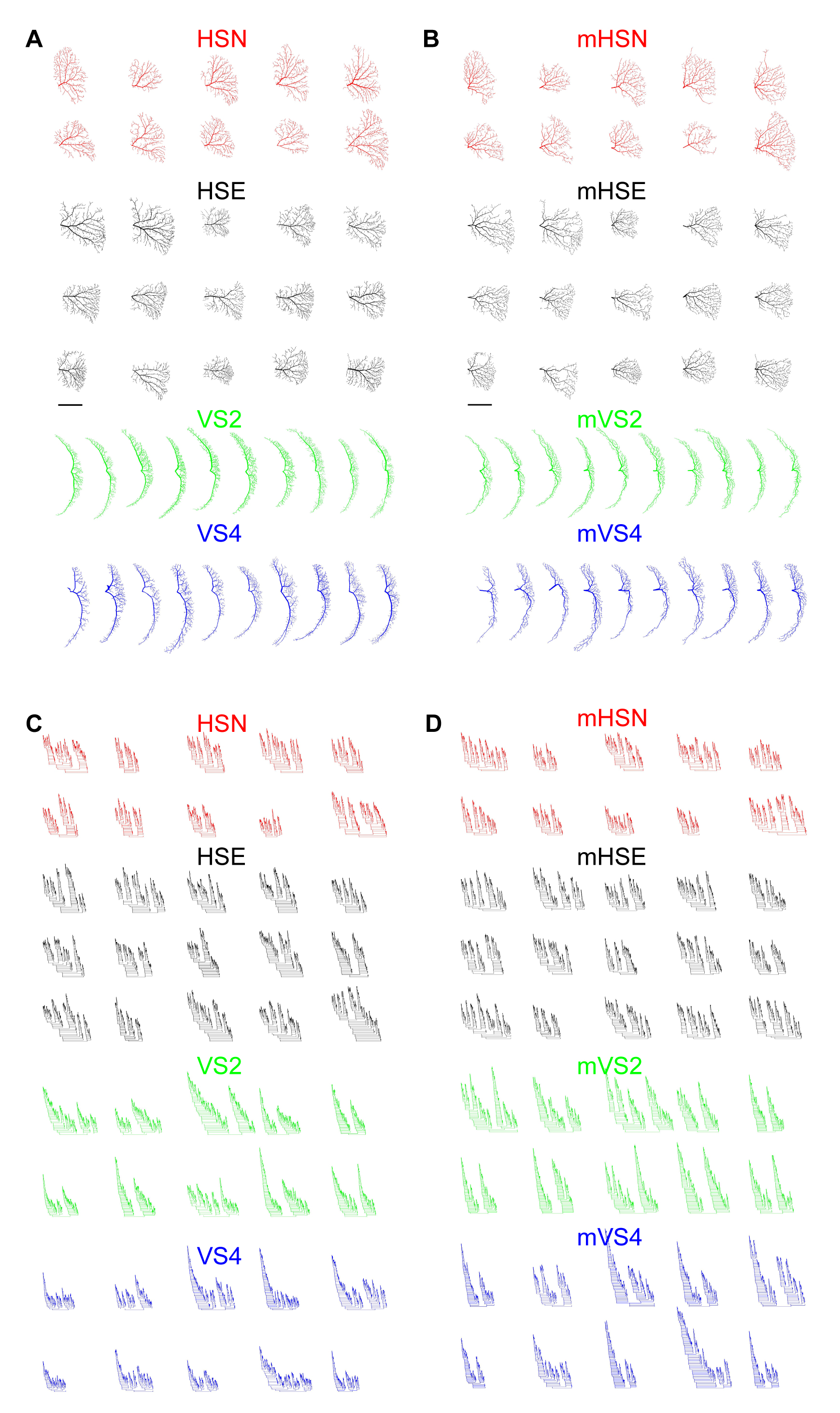

Supplement: Figure S3 — Overview of all 45 reconstructed LPTC dendrites and their artificial correlates. (A) Real manual dendrite reconstructions. (B) All constructed artificial LPTC dendrites. The model dendrites were grown in the spanning fields displayed in (A) in the same order. Diameter tapering was mapped here onto the branching structures for visual aesthetic purposes [12]. The artificial dendrites are hard to distinguish from their biological counterparts. (C, D) Overview of all dendrograms: comparison between reconstructed and artificial dendrites same colours as used in the main article. Dendrograms were sorted to put heavier trees (with larger sub-trees) on the left side. Although, taken one by one, dendrograms of artificial dendrites would not perfectly reproduce the corresponding partner (since they originated from random distributions of points), a trend of similarity is evident. This particularly illustrates how the differences in branching between HS and VS cells relates to the spanning fields of their dendrites since all artificial dendrites originate from the same branching rule. This is strongly in favour of a common branching rule for all cells. And this common rule is most likely very similar to the one applied to obtain the artificial dendrites. (2.02 MB TIF) [file pcbi.1000251.s003.tif]
